# Effect of Health Profession Students’ Community Engagement on Open Defecation-Free Status: A Quantitative Assessment of a Community-Led Total Sanitation and Hygiene Program in Southwestern Uganda

**DOI:** 10.7759/cureus.85858

**Published:** 2025-06-12

**Authors:** Moses Ntaro, JohnBosco Isunju, Fassou M Grovogui, Jonathan Izudi, Lenka Benova, Edgar Mulogo, John C Ssempebwa

**Affiliations:** 1 Community Health, Mbarara University of Science and Technology, Mbarara, UGA; 2 Disease Control and Environmental Health, Makerere University School of Public Health, Kampala, UGA; 3 Public Health, Gamal Abdel Nasser University of Conakry, Conakry, GIN; 4 Reproductive Health, Institute of Tropical Medicine in Antwerp, Antwerp, BEL

**Keywords:** community enagement, community led total sanitation, health extension works, health profession students, odf, open defecation, open defecation free status, students, uganda

## Abstract

Introduction

Uganda’s Community-Led Total Sanitation (CLTS) strategy is based on the use of health extension workers (HEWs) for its implementation at the community level. However, identification of other facilitators to improve and accelerate the scaling up of the CLTS intervention has not been explored. This study evaluated the effect of a student-led CLTS intervention on open defecation and open defecation-free status outcomes.

Methods

The study was conducted in two sub-counties in Kabale district in southwestern Uganda, Rubaya and Buhara. We used a pre-post study with a nonequivalent control group quasi-experimental study design to measure the effect of a student-led CLTS intervention on’ the levels of household open defecation (OD)-free (HODF) status. A total of six parishes from two study sub-counties with no main towns and having low latrine coverage were selected. A total of 50 villages were randomly selected, and 25 villages were assigned to each of the two study groups: (i) the student-led CLTS intervention group, and (ii) the conventional CLTS control group. To avoid contamination between the sub-counties, a buffer sub-county was left between. A total of 492 respondents from different households participated in the before and after CLTS intervention surveys.

Results

In the intervention group, the proportion of households practicing OD decreased (24.69% to 14.04%), and the households that obtained the HODF status also increased (2.88% to 6.14%). On the other hand, in the control group, OD increased (23.69% to 39.92%), and households with HODF status reduced (3.21% to 0.84%). The results showed that the households in the intervention group had higher odds of not practicing OD (OR 3.73; 95%CI: 1.01-13.77) compared to the households in the control group. Similarly, in the intervention group, households had a higher odds of attaining the HODF status (OR 13.20; 95%CI: 3.26- 53.55) compared to the control group.

Conclusion

The student-led CLTS reduced OD in the parishes where they worked. This study shows that other resource persons, such as health profession students, are a valuable resource that can fill in the gap in sanitation promotion activities. They will be able to successfully complement the already overburdened health workers, as in the case of Uganda.

## Introduction

The 2030 Agenda for Sustainable Development focuses on leaving no one behind in sanitation access. This ambitious agenda requires United Nations (UN) member countries to increase their efforts fivefold if the global target of sanitation access for all is to be achieved [1-3]. Worldwide, 3.5 billion people lack safely managed sanitation, with 419 million people still practicing open defecation (OD) [3]. OD has consequences for child mortality and development [4]. In addition, poor sanitation leads to fecal contamination of the environment, and together with poor hygiene practices such as hand washing, has resulted in an estimated 577,000 deaths annually [5,6]. More so, it’s estimated that two million children die annually due to diseases related to poor access to or quality of water or sanitation and suboptimal hygiene [7].

According to the 2015 UN General Assembly, poor sanitation conditions led the UN to reaffirm the importance of sanitation by prioritizing it in the Sustainable Development Goals (SDGs) as SDG 6 [8]. The goal aims to attain universal access to sufficient and equitable sanitation and hygiene for all, while eradicating OD by 2030, with a focus on initiatives that emphasize community participation.

In low- and middle-income countries (LMICs), for many years, governments and their development partners, like nongovernmental organizations (NGOs), have developed strategies to improve sanitation, such as providing free or subsidized latrine facilities to communities. Also, hygiene and health education programs like Children’s Hygiene and Sanitation Training (CHAST) [9] and Participatory Hygiene and Sanitation Transformation (PHaST) [10] were regularly combined with latrine subsidies [11,12]. Even though these approaches increased latrine coverage and awareness of health benefits [11], sanitation professionals realized that they were limited to creating demand for latrines and behavior change among communities, and largely, they are believed not to have resulted in household OD-free (HODF) status [12-14]. An impact assessment study of a five-year water and sanitation improvement programme in Odisha, a state of India, revealed an increase in latrine coverage from 15% to 48%; unfortunately, in the same study it was established that among the households that had built a latrine, 39% of the adults and 52% of the children reported to still practice OD [15]. To address similar challenges of community failures in demand for latrines and behavior change, the Community-Led Total Sanitation (CLTS) approach was developed [16].

The CLTS methodology has three standard phases as delineated in the CLTS Handbook: pre-triggering, triggering, and follow-up [17]. The initial phase of pre-triggering commences with community engagement and endorsement by leaders. Triggering is a community gathering facilitated by an external moderator who uses participatory tools, such as a sanitation map, designed to elicit an emotional reaction and foster a collective aspiration within the community to improve the inadequate sanitation conditions. Subsequently, facilitators will conduct follow-up visits to the villages to assess progress and assist the community in eradicating OD. Primarily, health extension workers (HEWs) spearhead the adoption of CLTS following a five-day training program [17].

Generally, progress has been made globally through the CLTS approach. For example, some local government areas in Nigeria, some districts in Zambia, and some counties in Kenya attained OD-free (ODF) status following the CLTS interventions [18,19]. Moreover, the CLTS sanitation promotion strategy has so far been adopted in more than 50 LMICs, including Uganda [20]. A study in Mali provided new evidence that increased private latrine ownership has doubled among households in villages that received a CLTS intervention [19]. These findings justified the scale-up of the CLTS programme in rural Mali and implied that the CLTS approach can be effective in improving access to sanitation in rural areas [21]. The CLTS approach has also been associated with improvements in child growth because a CLTS programme reduces child exposure to fecal contamination through reduction in OD and improvement in hand washing behavior [21,22]. However, for CLTS to register the desired result, it depends on the baseline settings, such as a high baseline of OD and high social capital. In addition, for CLTS outcomes to be more sustainable, conditions such as an enabling environment of access to latrine products and materials and sufficient follow-up visits are required [8].

In most of the countries where CLTS has been adopted as a national sanitation strategy, such as Uganda, HEWs, such as health assistants, community development officers, and health inspectors, are tasked with the implementation and roll-out of the strategy. Unfortunately, most of these low-income countries are undergoing a global health workforce crisis of a shortage of health professionals in rural and remote areas [23]. Similarly, the Ngor declaration on sanitation and hygiene, signed by African ministers at AfricaSan 4, identified the sanitation and hygiene human resource gap. It also established that the government’s over-reliance on community volunteers has affected the scale-up and sustainability of sanitation outcomes. This realization led to the formulation of Ngor commitment 5 to focus on addressing this gap at all levels [24]. In the face of this Human Resource for Health (HRH) gap, it is not possible for many African countries to have adequate HEWs for sanitation and hygiene promotion and CLTS implementation. In addition, national and local resources are always insufficiently allocated to sanitation, and governments often overdepend on development partners such as NGOs and humanitarian agencies for short-term CLTS interventions in selected areas [24].

Therefore, with regard to the hygiene and sanitation improvement coverage raised by the SDGs, new strategies are required. This is because the earlier national strategies for CLTS did not account for the SDG shift and the Ngor Declaration on sanitation and hygiene, which focus on universal access to adequate and sustainable hygiene services within the next six years (2030). With this goal integrated into national policies and plans among African countries, the scale and pace will need to increase drastically if SDG 6.2 is to be attained [24]. This, therefore, will require innovative and appropriate strategies that will support and scale up the existing hygiene and sanitation promotion interventions such as CLTS.

Some studies have been conducted to evaluate the performance of different actors in CLTS implementation. Such studies provide very useful information to guide the scale-up of the CLTS strategy. In a study conducted in Ethiopia, teachers were compared to HEWs in CLTS facilitation. The findings of this study revealed that teachers were less effective compared to the HEWs. This occurred due to teachers' additional responsibilities and a preliminary absence of support from local officials [6]. Interestingly, in the second year following completion of the CLTS intervention, the less effective teacher, facilitated CLTS caught up with the HEW-facilitated CLTS in sustaining CLTS outcomes [6]. In another related study in Ghana, natural leaders from the community were selected and trained in participatory, social, and technical skills during a HEW-facilitated CLTS intervention, which substantially improved the CLTS outcomes [25]. These studies provide evidence that CLTS outcomes can be sustained in the short term when interventions include training and building capacity for local actors [26]. Therefore, identifying appropriate actors in order to maximize CLTS outcomes is still a priority in CLTS implementation and scale-up.

Educators in medical schools acknowledged the central role in training to better serve and be accountable to communities. This is because social accountability is increasingly becoming an indispensable role for medical schools. Medical schools are required to contribute to improving the health status of the local communities and contribute to the “Health for All” strategy [23,27,28]. Therefore, many health professional training institutions globally have integrated Community-Based Education (CBE) into the training curricula [29]. The CBE curricula demand that health professional students have part of their learning within communities. It exposes medical students to realities in healthcare in less privileged communities. Gradually, medical education training has further evolved to student community engagement programs such as Community Engaged Medical Education (CEME), which stresses an interdependent and reciprocal relationship between the health profession training institutions and the community served. It envisages that community people are not just observers or recipients providing the settings for medical education training, but are also engaged in the education and services to provide solutions to their health problems in the community. Therefore, students' experiential learning objectives and activities are fundamentally aligned to priority community health care needs, and the student is the center of partnership between the community and the medical school for improving community health status [23]

Studies have shown that community-based education (CBE) programs improve students’ abilities to serve communities. It also increases their empathy, together with communication skills [30]. Most studies that have been done on students involved in CBE programmes have focused on the effect of learning activities in communities on students, such as students’ willingness to practice in a rural setting [31-33]. On the other hand, in a few studies looking at the contribution of students among host communities, they have revealed that students support the delivery of healthcare in health facilities and provide health education to communities [34,35]. These studies further recommend the need for a comprehensive examination of CBE programs in different settings in order to further quantify and document the contributions of health profession students [34].

A study by the consortium of medical schools in Uganda showed that improved malaria prevention and treatment-seeking behavior among parents of children under five years was associated with CBE presence in their communities [29]. In a related study, students participating in the CBE program provided health talks and demonstrations that increased awareness of behaviors and disease prevention in the communities. Students achieved this through engaging with the local communities in activities such as home visits and health workshops [36].

Mbarara University of Science and Technology (MUST), located in South Western Uganda, was established in 1989 and is based on the CBE philosophy. Its oldest faculty, the Faculty of Medicine, has been running a CBE programme since its inception. Annually, health profession students conduct health education activities among CBE host communities. In the CBE student host communities in South Western Uganda, sanitation and hygiene remain a major challenge, with more than half of the households (55%) using unimproved pit latrines. Generally, its sanitation status is more like that at the national where about 8.7% (22.4 million people) practice OD, and many villages are not ODF [37].

The Uganda Ministry of Health is implementing the Uganda Sanitation Fund (USF) programme funded by the Global Sanitation Fund (GSF) with the aim of eliminating OD to attain ODF communities [38]. To achieve the purpose of the USF programme, four outcomes were agreed upon, which, among others, include identifying innovative sanitation and hygiene approaches that are scalable. Following its five years of implementation in 44 out of 102 districts using the CLTS strategy, 78% of the villages have been triggered, and 37% of the villages have been certified as ODF [39]. Despite the achievements, the programme that is mostly donor-funded remains with challenges of further scaling up and sustaining the CLTS intervention [40]. Therefore, there is a need to identify and innovate approaches that will complement the Ministry of Health's effort to scale up and sustain the CLTS intervention. It’s in this line that we proposed investigating the contribution of student community engagement in the CLTS implementation to attain ODF status is an innovation in the above direction. This study, therefore, fills the knowledge gap on the use of health profession students in the CBE program as new actors to facilitate the CLTS intervention and examine their effect on household ODF status.

Therefore, this study evaluated the effect of a student-led CLTS intervention by comparing OD and ODF status outcomes between intervention villages (villages that received the student-facilitated CLTS) and control villages (which received routine sanitation intervention by the HEWs).

## Materials and methods

This study had a pre-post structure with a non-equivalent control group quasi-experimental study design. It was conducted in two subcounties (Rubaya and Buhara) of Uganda from March 2021 to April 2023. The study was approved by the Higher Degrees Research Ethics Committee, School of Health Sciences, College of Public Health, Kampala, Uganda. It was registered with the Uganda National Council of Science and Technology (registration number HS1135ES).

Study population

**Figure 1:**
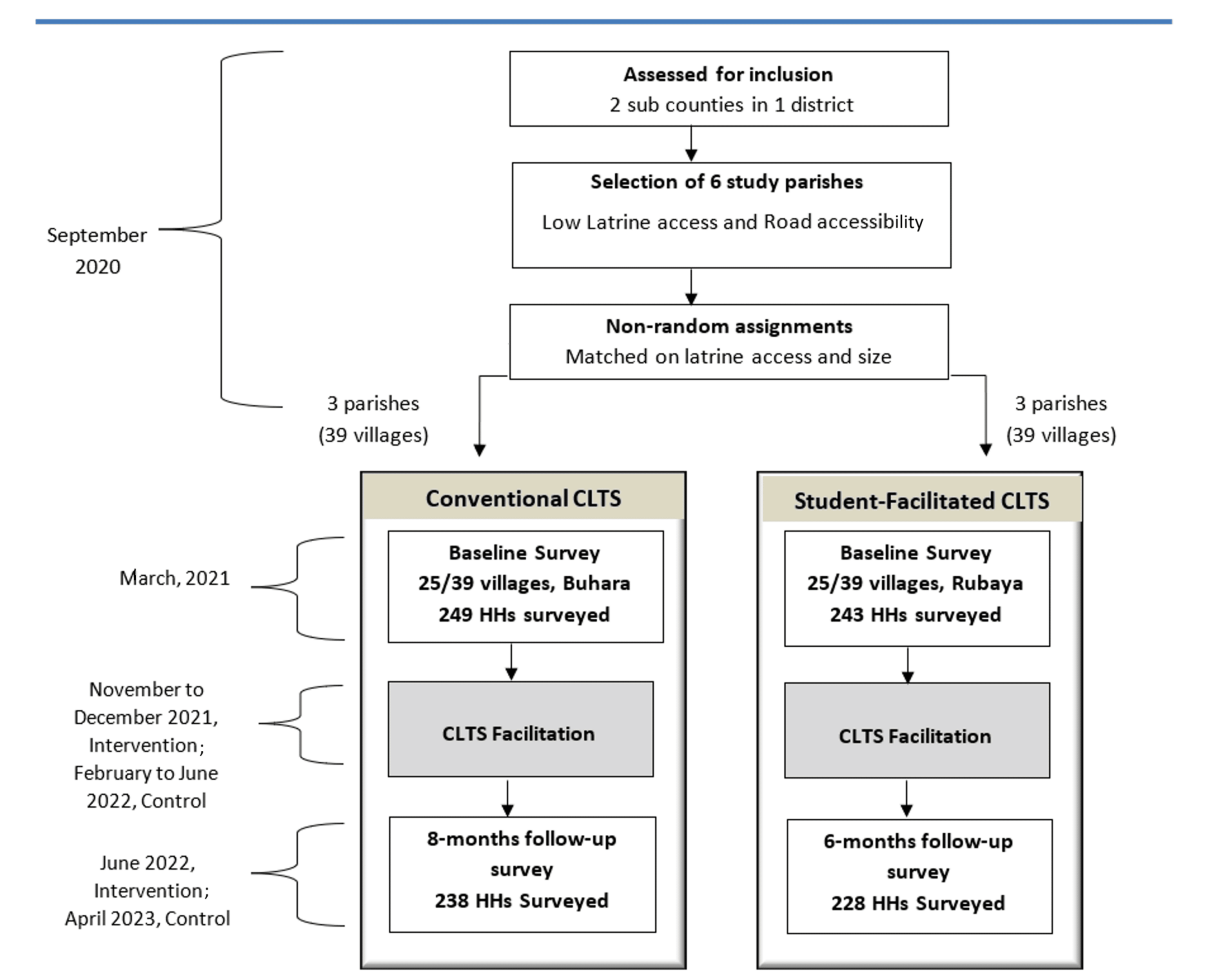
Timeline and sequence of the pre-post quasi-experimental study design Adapted from: Crocker et al., 2016 [6] CLTA: Community-Led Total Sanitation; HH: household

Kabale district was selected because it hosts most of the Mbarara University of Science and Technology (MUST) students on their student community engagement placement. Two subcounties (Rubaya and Buhara) with poor sanitation indicators were selected. In Uganda's decentralized government structure, both the district and subcounty possess political authority and financial autonomy [41]. The district local governments supervise and support the subcounty local governments [41]. Within the subcounties, a total of six road-accessible parishes with no main towns and having low latrine coverage according to the subcounties and district reports were selected [42]. These were pre-matched based on latrine accessibility and parish size (number of households). We manually designated them to receive either conventional or student-facilitated CLTS (Figure 1). The pre-matching was done in order to minimise bias and reach closer to random assignment to attain similar comparison groups. We used pre-matching because it is an established, valuable tool for evaluating community demand-driven sanitation policies [43].

Each parish has approximately 13 villages. Then we assigned randomly 25 selected villages in the three parishes of Rubaya subcounty to receive the student CLTS intervention, and the other 25 randomly selected villages in Buhara to receive the conventional CLTS. To avoid contamination between the intervention and the control villages, the subcounties of Rubaya and Buhara had a buffer subcounty between them.

CLTA intervention

The CLTS intervention consisted of three standard stages as explained in the CLTS Handbook [17]. The CLTS concentrated on a single day of ‘triggering’ and two follow-up visits that were referred to as post-triggering follow-up visits. This bottom up community sanitation approach involved health profession students who facilitated entering the selected villages and, by using a selection of tested community participatory techniques, elicited emotions such as shame, embarrassment and disgust among community members during village meeting to help community members realize that by practicing OD they are always feeding on each other’s feces. This realization was designed to bring about a transformation in the villages as the village members made strong decisions to come up with a plan to stop OD.

Health Profession Student-Led CLTS

The community engagement program known as the CBE Research and Service (COBERS) under the Faculty of Medicine at MUST organized its annual student engagement course, referred to as the Leadership and Community Placement (LCP) course, for undergraduate health profession students. The undergraduate health profession students who undertake the LCP course are fourth-year medicine, third-year nursing science, third-year medical laboratory sciences, third-year physiotherapy, second-year pharmacy, and first-year pharmaceutical sciences. A total of 355 students were divided into interdisciplinary student teams of about 7-12 students and placed across 23 urban and 10 rural communities by the COBERS program. While in these communities, students supported by a university supervisor undertook a community diagnosis and designed a low-cost intervention, mostly educational, to address the priority community challenge. One of these groups, comprising 12 students, was selected, trained, and prepared to participate in the CLTS intervention in the rural placement community in Rubaya sub-county, Kabale district.

Training of the student group: The training for the selected 12 students on how to facilitate CLTS was done in their first week of the three-week community engagement program. The four-day training was delivered by the Kabale district CLTS lead trainer, who was one of the district’s health workers. Although the training was a day less than the standard five days, it was similar to that provided to the HEWs facilitating CLTS within the district.

The 12 students were divided into four teams, comprising three students per team. Student teams were briefed by the health inspector in charge of their operation area before engaging in the CLTS activity. Since there were two HEWs in the sub-county trained in CLTS facilitation, they were expected to work along with the student teams; however, they only managed to attend two to three triggering sessions. The four student teams implemented CLTS in the 25 villages within three weeks of community engagement. The students came up with a work plan and triggered all 25 villages, and also did a post-triggering follow-up visit in each village. They also facilitated the selection of natural leaders to form village sanitation committees in each village. Details of the activities that students did during the CLTS implementation in every village were provided in village CLTS implementation reports. A month after the triggering and community placement, students travelled to these villages and, with guidance from the HEWs, conducted a post-triggering follow-up. During this follow-up, students visited an average of 10 homes, reinforcing the sanitation and hygiene promotion messages and practices aimed at making households attain an ODF status using a checklist.

Pre-triggering, triggering, and post-triggering approaches used by the students: Student teams conducted pre-triggering community entry visits to their respective villages within a period of two days. In addition to informing the community leaders of the objectives of the visit, it also included setting the dates for the triggering exercise in the villages. Triggering employed the steps and techniques of Kar and Chambers [17] that have been adopted locally into the CLTS strategy within the country. The steps they followed are given below.

Step one was the defecation area transect walk/walk of shame. During this transect walk, students, together with the community members, walked through the village locating OD areas. They ensured that community members stand in OD areas inhaling the unpleasant smell and in the discomfort of the sight of feces/shit. This disgusting sight experience and the smell accompanied by visitors to their community were aimed at triggering community mobilization, as more people gathered to see what was happening. Feces identified during the transect walk were carried to the meeting point to be used during the demonstrations.

Step two was the trigger for disgust. Following the transect walk, students, together with the community members, congregated at the meeting point to conduct demonstrations about the oral faecal transmission routes. Two demonstrations were often performed. One was the water bottles demonstration to show contamination of drinking water with feces, and the second was food contamination using a piece of cake. During the water contamination demonstration, a grass leaf was always picked and made to touch the collected faeces, and then stirred in the water in the bottle. The bottle could be shaken to ensure that the faeces introduced are barely seen. Later, the water could be offered to one of the community members to drink, who would always completely refuse. During the food contamination demonstration, a piece of cake on a plate would be placed next to the feces. This would attract flies from the faeces to the cake. The contaminated cake would be offered to someone, too, who would also refuse it. At this point, students with the aid of the F-diagram would show community members the different agents and pathways that make faeces get to the mouth.

The third step was the calculation of the amount of feces and medical expenses. Finally, students facilitated families to calculate the feces they produce in a year, and also the medical expenses related to fecal-related diseases. This brought the ignition moment with the community members realizing that they have been eating each other’s faeces and then deciding on the collective solution of ending OD. Sanitation committees were formed, and their names were written down; students agreed with the committees on the plans for the follow-up.

The post-triggering follow-up took the USF's approach called the Follow-up MANDONA. MANDONA refers to an action-oriented community follow-up approach that aims at motivating communities to adapt simple, immediate, doable actions (SIDAs) to drive towards attaining an ODF status. The students conducted two follow-up visits, with the first one a few days after triggering to support communities in attaining the ODF status.

Finally, the verification and certification of ODF villages was expected to follow the ongoing process of villages first declaring themselves ODF, then followed by certification from the authorities, which was not part of the scope of our study. 

The CLTS by HEWs in the Control Villages

In the 25 control villages in Buhara subcounty, HEWs implemented CLTS using the local government guidelines. Since CLTS is the sanitation promotion strategy used in Kabale, similar steps of pre-triggering, triggering, and post-triggering follow-up were used by the HEWs. The CLTS implementation by HEWs was led and supervised by the district principal health inspector. The principal investigator of the current study received a report from the principal health inspector about the CLTS implementation process that indicated that the activity took five months.

Eligibility criteria

All sampled households that had the heads of the household or their spouses at home during the household data collection exercise were included in the study. Sampled households that had very old household heads or their spouses who were unable to respond to the questionnaire were excluded from the study. In addition, any eligible individual who was deemed to be mentally unwell or under the influence of drugs or alcohol was excluded from the study. Finally, only those who provided consent were included in the study.

Sample size estimation

Since we were interested in a proxy indicator of a health outcome, we calculated the sample size necessary to measure the effect of change in OD. In addition, we inflated the calculated sample size because our study units were villages receiving the intervention, although our primary outcomes were measured on individuals in the households nested within those villages. Using the sample size calculation formula for studies using clusters [44], the sample size (m) was inflated by the design effect, and intracluster correlations (ICC) were accounted for. We assumed power of 80%, 5% confidence two-sided, and ICC (ρ) of 0.12 and design effect (DE) = [1+ n-1]ρ. The formula used was: $m = \left( \frac{(Z_{1-\alpha/2} + Z_{1-\beta})^2 \left[ P_1(1 - P_1) + P_2(1 - P_2) \right]}{D^2} \right) \times \left[1 + (n - 1)\rho \right]$, where, n is the number of individuals per cluster, which is approximately 70; P_1_ is the proportion of households with ODF status in the control arm, which is 0.2; P_2_ is the proportion of households with ODF status in the intervention arm, which is 0.4; D is the approximate difference in OD between the intervention and control arms, which is 10%. Therefore, the total sample size for each arm (control and intervention) = 210. Considering a 5% loss to follow-up (210 x 1.05), we calculated a sample size of 220 in each group.

However, for intervention studies involving clusters, where n individuals are in each cluster. Then, C, the number of clusters required, was determined using the formula: $c = 1 + \frac{(Z_{\alpha/2} + Z_{\beta})^2 \left[ \frac{\pi_0(1 - \pi_0)}{n} + \frac{\pi_1(1 - \pi_1)}{n} + k^2(\pi_0^2 + \pi_1^2) \right]}{(\pi_0 - \pi_1)^2}$

Here, k is the coefficient of variation of the true proportions between clusters within each group. Previous studies show that k is < 0.25 [45].

The study area comprised villages (clusters) with approximately 70 eligible participants distributed among 70 households. The intervention was delivered in villages. Before and after the intervention, surveys were conducted to measure the proportion of households with ODF in the two arms. We proposed a K, conservative value of 0.12 [43] and a 10 pp. difference between intervention and control [6]. The ODF status proportion was estimated at 0.03. Therefore, to determine the number of villages (clusters) required in each arm, we assumed that the proportion of ODF status in the control villages increased to 0.2, ∏o=0.2 and that we required 80% power (zβ=0.84) to detect a significant difference (P<0.05: Zα/2=1.96) if the intervention increases the ODF status by 37% to 40%, ∏1=0.4. As earlier determined n, 70 is the number of eligible participants in each cluster. Therefore, the number of clusters (villages) c, required in the treatment group, was calculated using the second formula above, which is 25. To obtain households to participate in the study, a list of households was compiled for all the selected villages, and an average of 10 households per village was randomly sampled proportionate to the village population.

Six parishes were sampled from each of the two study subcounties in Kabale district. All the villages in the parishes were listed, and 50 villages were randomly selected. An average of 10 households were selected from each village. This provided a sample size of 492 respondents for the baseline survey and 468 for the follow-up survey that targeted the same households that were surveyed at baseline (Table 1). 

**Table 1:** Survey sampling counts for parishes, village, and household levels, by CLTS intervention and subcounty in Kabale district CLTS: Community-Led Total Sanitation; HEWs: health extension workers

			Sampled villages and households		Number of households surveyed	
CLTS Intervention groups	Subcounties	Parishes	Number of Villages	Number of households	Before intervention	After intervention
Student-Facilitated CLTS	Rubaya	Rwanyana	6	60	59	56
		Kibuga	6	60	60	51
		Karujanga	13	130	124	121
Conventional HEWs-Facilitated CLTS	Buhara	Rwene	7	70	70	63
		Kitanga	8	80	79	74
		Kafunjo	10	100	100	103
Total			50	500	492	468

Data collection

The household surveys and observations of household sanitation and hygiene were completed at the baseline (March 2021), and the follow-up surveys in the intervention area were conducted in June 2022 and in control villages in April 2023. The differences in the time for the interventions in the two groups were solely based on the activity plans for the two implementing institutions (the District Health Office, Kabale, and the Community Engagement Unit at MUST). Household surveys collected data on demographics, sanitation, hygiene, interactions, and recall of CLTS activities. Sanitation outcomes were assessed by observing OD and ODF status at the household [46]. Information was then collected from heads of households or their spouses about contextual and behavioral factors influencing latrine use and handwashing practices. These included the economic status of the household, which was estimated by collecting information on household effects such as radio ownership, television ownership, type of materials used for latrine construction, and amount of funds used for latrine construction. Other variables included age, household size, educational level attained, cleanliness of latrine, status of latrine, distance of latrine, past diarrhea illness, knowledge about health risks, distance to water source, type of water source, baseline OD, and latrine use. In addition, using the RANAS (Risks, Attitudes, Norms, Abilities, and Self-regulation) model factors, information on behavioral factors such as risks, attitudes, norms, abilities, and self-regulation was collected. 

The questionnaire tool used was adopted from Crocker et al. [6] and Harter et al. [25]. The questionnaire was translated from English into Rukiga. The translated questionnaire was utilized in interviewer training and examined by the first author and interviewers to confirm that the meaning of the questions remained unchanged. The interviewers conducted a pretest of the questionnaire in non-study areas with 11 households to verify content validity, language suitability, and question clarity. The survey utilized the mobile data collection software KoBoCollect v2021.3.4 (Kobo, Cambridge, Massachusetts, United States) [47], installed on tablet devices, and had a duration of approximately 45-60 minutes.

Data analysis

Descriptive statistics from household surveys and observations were used to establish differences between the intervention and control groups at baseline. The primary outcomes were changes in OD levels and ODF status in households. The OD and ODF status were established through observations at the latrine and household premises as described earlier. The difference-in-difference (DID) estimator was used to determine the level of change in households' OD and ODF status between the intervention and control groups, taking into account the baseline differences. A logistic regression model was built with OD and ODF status outcomes as the dependent variables. The analysis included data collected at two time points, that is, before and after the CLTS interventions. OD and ODF status were modeled using a logistic regression as a function of treatment group, time, and eight covariates (presence of latrine with superstructure, household spent above $16 on latrine building, absence of waste around the yard, respondent married, household visited by extension health worker in past two months, respondent attended CLTS triggering in past two months, household visited by the village health team (VHT) in past two months, household visited by students in past two months). The “svyset” command in STATA/BE 17 (StataCorp LLC, College Station, Texas, United States) was utilized to account for unequal selection probability, non-response rates, and village clustering of outcomes.

Although pre-matching was done and a DID estimator, together with a multivariable regression, was performed to cater for baseline differences between the groups, outcomes of interest were not solely attributed to interventions since our study design did not involve nonrandom assignment.

## Results

Baseline statistics

The two groups were similar in most characteristics, except that there were more married respondents, spending more than $16 in building latrine, more households with clean compounds, fewer households with latrine superstructure, and more reporting of participation in community engagement activities were observed in the intervention group (Table 2). More so, the primary outcomes, OD and HODF status, were not different in the two groups. Although pre-matching eliminated most of the sanitation difference, it did not completely eliminate the household latrine ownership difference at baseline (Table 2).

**Table 2:** Respondent and household characteristics at baseline in the control and intervention groups The respondents were heads-of-household or their spouses. The chi-square test was used to check for statistical differences between the Control and Intervention groups. ^v^ surveyor-observed; the remaining were self-reported by respondents ODF: open defecation free; CLTS: Community-Led Total Sanitation; VHT: village health team

Characteristics	Category	Study Groups		p-value
		Control (Convention CLTS)	Intervention (Health profession students-facilitated CLTS)	
Age of respondents (in years)	Mean (SD)	34.6 (19.7)	32.9 (18.9)	0.340
Gender of respondents, n (%)	Female	172 (51.7%)	161 (48.3%)	0.500
Marital status, n (%)	Married	213 (48.7%)	224 (51.3%)	0.019
Household size (people), n (%)	> 7	226 (49.9%)	227 (50.1%)	0.280
Education level, n (%)	Primary	135 (50.0%)	135 (50.0%)	0.700
Economic status, n (%)	Owns radio	150 (48.7%)	158 (51.3%)	0.270
	Owns a television screen	14 (42.4%)	19 (57.6%)	0.330
	metal roofing material	249 (50.6%)	243 (49.4%)	1.00
	Spent above USD 16 on latrine building	93 (46.5%)	107 (53.5%)	0.010
Open defecation, n (%)	Feces observed in the household vicinity^v^	59 (49.6%)	60 (50.4%)	0.800
Household ODF status, n (%)	Attained ODF status^v^	8 (53.3%)	7 (46.7%)	0.830
Compound cleanliness status, n (%)	Waste present around the yard^v^	202 (54.4%)	169 (45.6%)	0.003
Latrine with superstructure, n (%)	Absence of latrine with superstructure^v^	15 (28.8%)	37 (71.2%)	< 0.001
Latrine sharing, n (%)	Uses shared latrine	33 (49%)	34 (51%)	0.120
Latrine status during rainy season, n (%)	Latrine unusable	19 (38.8%)	30 (61.2%)	0.081
Handwashing station, n (%)	Presence of handwashing^v^	36 (43.4%)	47 (56.6%)	0.150
Water source, n (%)	Use of improved water source	124 (49.0%)	129 (51.0%)	0.740
Time around water collection, mean±SD	Walking time to water source (minutes)	18.4 ±29.3	21.1 ±31.6	0.330
Category of latrine, n (%)	Improved pit latrine	9 (50.0%)	9 (50.0%)	0.930
Hygiene education provided, n (%)	Hygiene education within past 2 months	162 (47.9%)	176 (52.1%)	0.078
Extension worker home visit, n (%)	Visited by extension health worker in past 2 months	13 (31.7%)	28 (68.3%)	0.011
Participated in CLTS triggering, n (%)	Attended CLTS triggering in past 2 months	14 (26.9%)	38 (73.1%)	< 0.001
VHT home visit, n (%)	Visited by VHT in past 2 months	131 (46.6%)	150 (53.4%)	0.041
Students' home visit, n (%)	Visited by health profession students in past 2 months	2 (16.7%)	10 (83.3%)	0.020

Sanitation outcomes

Following the implementation of CLTS in the intervention and control groups, that is to say from the baseline to follow-up, the proportion of households practicing open defecation decreased (24.7% to 14.0%). Also the households that obtained the ODF status increased (2.9% to 6.1%) only in the intervention group. In the control group, the open defecation increased (23.7% to 39.9%) and households with the ODF status reduced (3.2% to 0.8%).

There were significant differences in most of the sanitation characteristics after the CLTS implementation, with the intervention group recording better results. Apart from the proportion of households with handwashing materials at the handwashing station being higher in the control group, most of the other good sanitation characteristics were better in the intervention group (Table 5). The difference-in-difference estimator was performed using open defecation and open defecation-free status, modeled using a logistic regression as a function of treatment group, time, and eight covariates that were statistically significant at baseline. The results showed that the households in the intervention group had a higher odds of not practicing open defecation (3.73; 95%CI: 1.01 - 13.77) compared to the households in the control group. Similarly, in the intervention group, households had more odds of attaining the open defecation free status (13.20; 95%CI: 3.26- 53.55) compared to the control group (Table 5).

Health promotion and CLTS activities by HEWs and students

Before the intervention, households in the student-CLTS facilitated parishes reported having received more household visits from HEWs, VHTs, and students. They had also participated more previously in CLTS triggering compared to the conventional CLTS parishes. However, this does not affect the intervention effect estimates presented earlier because these baseline community engagement activities were included as covariates in the outcome regression models. After the intervention, the findings showed that household visits by HEWs, VHTs, and students increased more drastically in the student-CLTS facilitated parishes, and the increases were more statistically significant (Table 3).

**Table 3:** Health promotion and CLTS activities pre-post by HEWs and students The respondents were heads-of-household or their spouses. The chi-square test was used to check for statistical differences in community engagement activities. Data was self-reported by respondents. CLTS: Community-Led Total Sanitation; HEWs: health extension workers; VHT: village health team

Time	Variable	Variable Description	Control Group, n (%)	Intervention Group, n (%)	p-value
Baseline	Hygiene education provided	Had hygiene education with with past 2 months	162 (47.9%)	176 (52.1%)	0.078
	Extension worker home visit	Visited by HEW in past 2 months	13 (31.7%)	28 (68.3%)	0.011
	Participated in CLTS triggering	Attended CLTS triggering in past 2 months	14 (26.9%)	38 (73.1%)	< 0.001
	VHT home visit	Visited by VHT in past 2 months	131 (46.6%)	150 (53.4%)	0.041
	Students' home visit	Visited by health profession students in past 2 months	2 (16.7%)	10 (83.3%)	0.020
Follow up	Hygiene education provided	Had hygiene education with with past 2 months	201 (49.3%)	207 (50.7%)	0.038
	Extension worker home visit	Visited by HEW in past 2 months	31 (34.8%)	58 (65.2%)	<0.001
	Participated in CLTS triggering	Attended CLTS triggering in past 2 months	39 (28.3%)	99 (71.7%)	< 0.001
	VHT home visit	Visited by VHT in past 2 months	103 (40.1%)	154 (59.9%)	<0.001
	Students' home visit	Visited by health profession students in past 2 months	16 (13.0%)	107 (87.0%)	<0.001

There were significant differences in most of the sanitation characteristics after the CLTS implementation, with the intervention group recording better results (Table 4). Apart from the proportion of households with handwashing materials at the handwashing station being higher in the control group, most of the other good sanitation characteristics were better in the intervention group. 

**Table 4:** Respondent household sanitation characteristics after CLTS interventions in the control and intervention groups CLTS: Community-Led Total Sanitation; ODF: open defecation free

Characteristics		Control Group (n=238), n (%)	Intervention Group (n=228), n (%)	p-value
Presence latrine with superstructure	Yes	155 (41.2%)	221 (58.8%)	<0.001
Presence of hole cover	Yes	65 (43.6%)	84 (56.4%)	0.027
	No	173 (54.6%)	144 (45.4%)	
Evidence of latrine use	Yes	198 (50.4%)	195 (49.6%)	0.49
	No	40 (54.8%)	33 (45.2%)	
Use of handwashing facility	Yes	55 (62%)	34 (38%)	0.027
	No	29 (44%)	37 (56%)	
Presence of handwashing facility	Yes	84 (54.2%)	71 (45.8%)	0.34
	No	154 (49.5%)	157 (50.5%)	
Presence of material at the handwashing station	Yes	64 (67%)	31 (33%)	<0.001
	No	20 (33%)	40 (67%)	
Availability of water at the handwashing station	Yes	58 (48.7%)	61 (51.3%)	0.013
	No	26 (72.2%)	10 (27.8%)	
Open defecation	Observed	95 (74.8%)	32 (25.2%)	<0.001
	Not Observed	143 (42.2%)	196 (57.8%)	
Household ODF status	Yes	2 (12.5%)	14 (87.5%)	0.002
	No	236 (52.4%)	214 (47.6%)	
Compound cleanliness status	Waste present	161 (46.8%)	183 (53.2%)	0.002
	Waste absent	77 (63.1%)	45 (36.9%)	
	latrine usable	226 (51.6%)	212 (48.4%)	
Share latrine	Yes	66 (68.8%)	30 (31.3%)	<0.001
	No	165 (45.5%)	198 (54.5%)	

The difference-in-difference estimator was performed using OD and ODF status modeled using a logistic regression as a function of treatment group, time, and eight covariates that were statistically significant at baseline. The results showed that the households in the intervention group had higher odds of not practicing open defecation (3.73; 95%CI: 1.01-13.77) compared to the households in the control group (Table 5). Similarly, in the intervention group, households had more odds of attaining the open defecation free status (13.20; 95%CI: 3.26-53.55) compared to the control group 

**Table 5:** Difference-in-difference logistic regression parameters: open defecation and open defecation free status as a function of the intervention group compared to control group in Kabale district. CLTS: Community-Led Total Sanitation; VHT: village health team; HEW: health extension worker

Variable	Open Defecation		Open Defecation Free Status	
	P-value	Adjusted Odds Ratio (95%CI)	P- value	Adjusted Odds Ratio (95%CI)
Intervention	0.814	1.09 (0.51 - 2.31)	0.495	0.61 (0.14-2.63)
Time	0.91	0.09 ( 0.24 – 4.86)	0.483	3.40 (0.10 – 117.60)
Intervention * Time interaction	0.048	3.73 (1.01 – 13.77)	0.001	13.20 (3.26– 53.55)
Presence of latrine with superstructure	0.022	2.46 (1.15 – 5.25)	N/A	N/A
Household spent above $16 on latrine building	0.960	1 (0.70 – 1.46)	<0.001	2.89 (1.86 – 4.48)
Waste absent around the yard	0.843	1.05 (0.65 – 1.68)	0.007	4.37 (1.56 – 12.19)
Respondent married	0.624	0.83 ( 0.40 – 1.77)	0.791	1.32 (0.15 – 11.66)
Household visited by HEWs in past 2 months	0.822	0.90 (0.32 – 2.47)	-	-
Respondent attended CLTS triggering in past 2 months	0.323	0.69 (0.32 – 1.48)	0.483	1.61 (0.40 – 6.43)
Household visited by VHT in past 2 months	0.393	1.36 (0.66 – 2.81)	0.339	2.04 (0.45 – 9.22)
Visited by students in past 2 months	0.966	0.97 (0.29 – 3.30)	-	-

## Discussion

We found that health profession students have the ability to facilitate sanitation promotion at the community level. There have been no prior rigorous studies of health profession students leading a CLTS intervention during their mandatory academic community engagement program. Our findings are in agreement with a previous study by Atuyambe et al. that demonstrated effective contributions by students to healthcare delivery at health facilities and improved sanitation services at the community level [34]. In their study, they were able to qualitatively explore the student contributions expressed by the community leaders, members of the village health, and health facility leaders. However, by the nature of their study design, quantification of the student contributions towards sanitation improvements was not possible. In the current study, we have attempted to quantify the student contribution in sanitation promotion using more rigorous household sanitation indicators of OD and ODF status to further strengthen the evidence of the contributions of the community engagement programs of university health professions training schools. 

There was a threefold reduction in OD practice and a twofold increase in HODF status in the student-facilitated CLTS parishes (intervention group) compared to the conventional HEWs-facilitated parishes (control group). This finding of a reduction in OD in the intervention group is consistent with previous studies that have demonstrated the effect of CLTS in reducing OD and latrine utilization [6,12,21,48,49]; however, the reduction was not registered in the control group. Studies have also shown that communities have attained ODF status certification after CLTS implementation [42-52]. Most of the studies used clusters, such as villages, as the unit of measurement for open ODF status certification. Few studies have measured ODF status at the household level. Okolimong et al. [52] established a higher HODF status (35%) compared to the 6.1% that we have reported in the student CLTS-facilitated parishes. The lower percentage in this study can partly be explained by the very low, less than 3% of households with HODF status before the intervention, and the short time between the intervention and evaluation.

The increase of OD and reduction of the proportion of HODF status in the control group can be explained by the low proportion of households with latrines and HEW visits. Although the CLTS intervention is expected to trigger communities to construct low-cost latrines [53], our findings show that most of those who had latrines had used no money or less than $16 to build them. Secondly, according to the CLTS implementation report by the district health focal person who was responsible for the parishes in the control group, it was indicated that the activity took five months. It is more likely than not that HEWs implemented the CLTS hurriedly to meet the timelines of this study. It’s also likely that the two HEW workers who were charged with the CLTS implementation and follow-up activities also got engaged in other job demands, such as supervision of bed net distribution exercise, health inspections of markets, and workplaces, thus implying that all the community activities required, especially community follow-up activities after triggering, may have not been conducted adequately in the control group villages. This is also reflected in our baseline findings, which showed low household community engagement activities by HEWs in the same parishes. Crocker et al. agree that there are some areas where the HEWs are busy, and other CLTS facilitators should be identified to support them in CLTS implementation [6]. Other studies, while establishing the most important activities for successful CLTS implementation resulting in ODF communities, have ranked follow-up and monitoring activities first [54]. Hence, no effect of the CLTS intervention was seen in the control group villages at the time of the follow-up survey. However, we argue that since this is their routine work with subsequent follow-up activities beyond the study period, improvements in latrine coverage will be realized so that OD reduction and ODF status attainment are achieved. Nevertheless, these findings emphasize the importance of monitoring CLTS interventions in order to ensure that OD is eliminated and ODF status is realized in the effort to prevent and control diarrheal diseases.

Although our findings show a reduction of OD and an increase in household ODF status in the intervention group, we also recognize the contribution of other factors. Our results show that the intervention group had a higher proportion of latrines with superstructures, were slightly economically better, and had less dirty compounds. These factors were accounted for in the DID logistic regression models, and the presence of a latrine with superstructure remained statistically significant for the OD reduction. Also, in the model for HODF status, households that had spent $16 or more and had less dirty compounds had higher odds of attaining the ODF status. This underpins the importance of understanding enabling contextual factors in CLTS interventions to achieve the elimination of OD. Orgill-Meyer et al. similarly report that ODF cannot be sustained if there are no latrine facilities [55]. Therefore, it’s paramount that CLTS interventions be complemented with poverty-targeted subsidies to support households in constructing latrine facilities [56].

We also observed that community engagement activities at the household level increased very significantly in the parishes with students. This was expected since the students worked with the VHT team members in community mobilizations and follow-up activities. The two HEWs working in the subcounty that received the student intervention were expected to support and guide the students. However, it is important to note that HEWs' home visits further increased at the households in the villages covered by the intervention group. This could either be that they also participated in the follow-up activities after the students had triggered the communities, or it could even be for other interventions, such as bed net distribution, which was happening around the same time. Whether it is the latter or former, students triggered a higher household engagement of VHTs and HEWs that contributed to the positive sanitation results presented in this study. This agrees with the philosophy of COBERS and Community Engaged Medical Education that students can act as change agents and facilitate communities to solve their problems [57]. This study demonstrates the contribution of student-led intervention when aligned with district priorities.

Limitations

One limitation of this study was the nonrandom assignment of the parishes, although differences were controlled for. Few study sites were used, although these sites represent rural subcounties in the district. The study duration was limited to less than eight months, and only the immediate effects could be evaluated. However, this still provided findings that can be a basis for long-term follow-up. Finally, assessing the quality of CLTS was not done since it was out of the scope of this study. However, the mentioned limitations do not affect the results presented.

Strengths

The application of a DID estimator, along with the incorporation of baseline covariates that differ between the groups, reduces the likelihood of bias in the effect estimates of the intervention relative to the control in this study.

## Conclusions

Students were able to use the CLTS approach to reduce OD in the parishes with the support of the existing health infrastructure. This therefore demonstrates that students can be an important resource in supporting rural communities that may be facing problems in the availability of human resources in resource-constrained settings.

Existing CLTS programmes should look at harnessing other resource persons, such as students under the COBERS approach, to promote and facilitate communities towards achieving the ODF status in the different communities.

## Appendices

**Table 6:** Household survey questionnaire tool measuring open defecation status, social, economic, and psychosocial determinants for behavior change HEF: Health Extension Worker

Section 1: Identification		
1	Name of the Subcounty	≪subcounty≫
2	Name of the Parish	≪Community≫
3	Name of the Village	≪Village≫
4	Household ID	≪UniqueID≫
5	Date	2020 / __ | ___ / M D
6	Name of the Research Assistant	
Section 2: Demographics		
7	What is the sex of the respondent?	1. ◯ Male 2. ◯ Female
8	What is your age?	| ___ | ___ | years
9	Are you married?	1. ◯ Yes 2. ◯ No
10	What is the highest education level in school that you completed?	| ___ | ___ | Primary/Secondary/Tertiary
11	Observe: Does the house have a metal roof?	1. ◯ Yes 2. ◯ No
12	Observe: How clean is the household compound?	1. ◯ Abundant trash and solid waste strewn around the yard 2. ◯ Less than 10 pieces of trash or solid waste evident in the yard 3. ◯ No trash or waste; the yard is clean of any debris 4. ◯ Littered feces
13	How many years has your family? lived in this household?	| ___ | ___ | years
14	How many years have you lived in this community?	| ___ | ___ | years
• Before moving to the next survey question, check to make sure the response to question 13 is lower than or equal to the response to question 14. • If response 13 is higher than response 14, ask both questions again starting with question 14.		
15a	What do you think is the highest priority for your community?	• Do not read the answers. • Check only one response. • If multiple answers are given, ask them to pick the most important one. 1. ◯ Schools 2. ◯ Health facilities 3. ◯ Roads 4. ◯ Electricity 5. ◯ Water supply 6. ◯ Sanitation facilities 7. ◯ Hygiene or handwashing 8. ◯ Housing 9. ◯ Employment
15b		10. ◯ Other (specify):
16	Do you have a television in your house?	1. ◯ Yes (go to question 17) 2. ◯ No (go to question 18)
17	Can you show me the TV?	1. ◯ Shown 2. ◯ Not shown, or not able to show it
18	Do you have a radio in your house?	1. ◯ Yes (go to question 19) 2. ◯ No (go to question 20)
19	Can you show me the radio?	1. ◯ Shown 2. ◯ Not shown, or not able to show it
20	How many people normally live in this household?	| ___ | ___ | people List the first names and age starting with the oldest Name Age ……………………. ………….. ……………………. ……………. …………………… …………… …………………… …………… ………………….. …………….. ………………….. ……………… ………………….. ………………. ………………… …..……… ………………….. …………. ………………….. …………..
21	How many individuals are 18 years and above?	| ___ | ___ | people
22	How many individuals are between 5 years and 18 years?	| ___ | ___ | people
23	How many individuals are 5 years and below? (Cross check the answers with question 20)	| ___ | ___ | people
• Before moving to the next question, check that responses 21 + 22 + 23 = 20, if the responses do not match, ask them again starting with question 20 • If the answer to question 23 is 0, go to question 27		
24	In the last two weeks, how many of your children 5 years of age or younger have had diarrhea?	| ___ | ___ | children
• If the answer to question 24 is 0 go to question 27		
25	Was he/she taken to a health facility for treatment?	1. ◯ Yes 2. ◯ No
26	Was he/she given any medicine or rehydration solution?	1. ◯ Yes 2. ◯ No
27	Do you think people not using latrines are a health risk in your village?	1. ◯ Yes 2. ◯ No
28	Who do you think should bear the cost of improving sanitation in your village? • Do not read the answers. • Circle all responses. • After they have finished responding, ask “are there any more occasions?” • If the respondent indicates that (s)he does not know, do not probe for additional responses.	Family / household Local leadership Government (woreda, zone, region, and/or federal) NGOs / partners Other (specify):
Section 3: Water		
29a	What is the main source of drinking water for members of your household?	1. ◯ River/stream 2. ◯ Pond/lake 3. ◯ Open spring 4. ◯ Protected spring 5. ◯ Open well 6. ◯ Protected well 7. ◯ Tubewell/borehole 8. ◯ Rainwater harvesting 9. ◯ Public tap 10. ◯ Piped water into dwelling or yard 11. ◯ Water vendor 12. ◯ Bottled water
29b		13. ◯ Other (specify):
30	In the last two weeks, was water unavailable from this source for a day or longer?	1. ◯ Yes 2. ◯ No
31	Do you share this water source with other households?	1. ◯ Yes 2. ◯ No
32	Do you pay to use this water source?	1. ◯ Yes 2. ◯ No
33	If this source is not in your dwelling or yard, how long does it take to walk to it?	| ___ | ___ |___| minutes
34	Do you have to queue or wait to get water at this source?	1. ◯ Yes 2. ◯ No
35	How long do you typically have to wait to get water at this source?	| ___ | ___ |___| minutes
36	Is this water source usable year-round?	1. ◯ Yes (go to question 38) 2. ◯ No (go to question 37)
37a	Where do you get drinking water when your main source is not available?	1. ◯ River/stream 2. ◯ Pond/lake 3. ◯ Open spring 4. ◯ Protected spring 5. ◯ Open well 6. ◯ Protected well 7. ◯ Tubewell/borehole 8. ◯ Rainwater harvesting 9. ◯ Public tap 10. ◯ Piped water into dwelling or yard 11. ◯ Water vendor 12. ◯ Bottled water 13. ◯ Main source is always available
37b		14. ◯ Other (specify):
38	Do you currently treat your drinking water?	1. ◯ Yes (go to question 39) 2. ◯ No (go to question 40)
39a	How do you treat your drinking water?	1. ◯ Chlorination 2. ◯ Filtration with a ceramic device (such as a clay pot, or a candle filter) 3. ◯ Filtration with a biosand filter 4. ◯ Solar disinfection 5. ◯ Boiling 6. ◯ Chemical coagulant (such as aluminum salt or iron salt)
39b		7. ◯ Other (specify):
40	Do you store your drinking water?	1. ◯ Yes (go to question 41) 2. ◯ No (go to question 46)
41	May I see the container(s) where you store it?	1. ◯ Allowed 2. ◯ Not allowed
42	Is this container used only for storing drinking water?	1. ◯ Yes 2. ◯ No
43	Observe: Does the container have a wide or narrow mouth?	1. ◯ Wide mouth (more than 10 centimeters across) 2. ◯ Narrow mouth (less than 10 centimeters across)
44	Observe: Does the container have a spigot?	1. ◯ Yes 2. ◯ No
45	Observe: Does the container have a lid or fitted cover?	1. ◯ Yes 2. ◯ No
Section 4: Sanitation		
46a	Where do members of your family usually go to defecate? (circle only one response)	1. ◯ Bush, field, river, or pond (go to question 73) 2. ◯ Dig and bury (go to question 73) 3. ◯ Latrine at their own household (go to question 47) 4. ◯ Neighbor’s household (go to question 58) 5. ◯ Communal or public latrine (go to question 58)
46b		6. ◯ Other (specify): (go to question 58)
47	May I see the latrine you use please?	Latrine present Latrine absent
47b		1. ◯ Allowed (go to question 48) 2. ◯ Not allowed (go to question 49 )• If allowed to see the latrine, walk to the latrine with the respondent
48	Observe: Has the path to the latrine been walked on recently?	1. ◯ Yes (grass is trampled, wet footprints are visible, or the path has recently been cleared) 2. ◯ No
49	How much money did you spend to build this latrine?	| ___ | ___ |___ |___ | Ush.
50a	What did you buy to construct your latrine? • Circle all responses.	Cement
50b		Pre-made slab/squat plate
50c		Wood
50d		Sheet metal (for walls or roof, etc)
50e		Labor/help for digging or construction
50f		Other (specify):
51	How many total hours did it take your family to build this latrine?	| ___ |___ | hours
52	Did anyone besides your family help you build this latrine?	1. ◯ Yes (go to question 53) 2. ◯ No (go to question 55)
53a	Who helped you build this latrine? • Circle all responses.	Neighbors, other community members
53b		Village Health Team Members
53c		Parish leaders
53d		Subcounty local government
53e		Church, mosque, or other religious group
53f		NGOs/partners
53g		Other (specify):
54	For how many hours did they help you build your latrine?	| ___ |___ | hours
55	Do you plan on changing your latrine before the start of the next rainy season?	1. ◯ Yes (go to question 56) 2. ◯ No (go to question 58)
56a	In what way do you plan to change your latrine? • Circle all responses.	New slab/squat plate
56b		New walls
56c		New roof
56d		New door
56e		New/replacement latrine
56f		New/replacement pit
56g		Other (specify):
57a	Who will pay for the changes to your latrine? • Circle all responses.	Me, my family, or my household Members
57b		Neighbor or friend
57c		Community members, or chief
57d		An NGO or outside organization
57e		The government, Health Extension Worker, or other officer of water or Health
57f		The latrine will not cost any money
57g		Other (specify):
58a	What kind of latrine does your family usually use? • Circle only one response	1. ◯ Bucket toilet 2. ◯ Simple Pit latrine 3. ◯ Ventilated improved pit latrine (VIP) 4. ◯ Composting toilet 5. ◯ Pour flush toilet 6. ◯ Septic tank
58b		7. ◯ Other (Specify):
59	Do you share this latrine with other households?	1. ◯ Yes (go to question 60) 2. ◯ No (go to question 63)
60	How many households do you share this latrine with?	| ___ |___| households
61	Are these households where only relatives of yours live?	1. ◯ Yes (57) 2. ◯ No (56)
62	Is this toilet used by people that you do not know?	1. ◯ Yes 2. ◯ No
63	Can you use this facility at all hours of the day and night?	1. ◯ Yes 2. ◯ No
64	How much time does it take on average to get to the place you defecate?	| ___ | ___ |___| minutes
65	Do you have to queue/wait to use this latrine?	1. ◯ Yes (go to question 66) 2. ◯ No (go to question 67)
66	On average, how long do you have to queue/wait?	| ___ | ___ |___| minutes
67	Since the beginning of the rainy season, did your latrine become unusable?	1. ◯ Yes (go to question 68) 2. ◯ No (go to question 70)
68a	Why was it unusable? • Circle all responses.	Roof problems
68b		Slab problems
68c		Pit overflow
68d		No water in tank
68e		Flushing mechanism broke down
68f		Bowl overflow/clogged
68g		Pipe breakdown
68h		Other (specify):
69	During that period, how many weeks was it unusable?	| ___ |___| weeks
70	Do any members of your family defecate in the bush, field, or nearby river or lake when away from home?	1. ◯ Yes 2. ◯ No
70a	Ask for each member in question 20 if he or she defecates in the open Write the total number of open defecators in the household. Then also their names and ages	| ___ | ___ | people List the first names and age starting with the oldest Name ----------------Age ………………………. ………….. ………………………. ……………. ……………………… …………… …………………… …………… ………………….. …………….. ………………….. ……………… ………………….. ………………. ……………… …..……… ……………….. …………. ……………….. …………..
71a	The last time a child 5 years or younger in your house passed stool, where did he/she defecate? • If the household does not have any children below 5 years of age go to question 73	1. ◯ Used potty (go to question 73) 2. ◯ Used diaper (go to question 72) 3. ◯ Went in his/her clothes (go to question 72) 4. ◯ Went in house/yard (go to question 72) 5. ◯ Went outside the premises (go to question 73) 6. ◯ Used own sanitation facility (go to question 73) 7. ◯ Used public latrine (go to question 73) 8. ◯ Don’t know (go to question 72)
71b		9. ◯ Other (specify): go to question 72
72a	The last time a child in your house passed stool, where were his/her feces disposed?	1. ◯ Dropped into toilet ffacility 2. ◯ Buried 3. ◯ Garden 4. ◯ Solid waste/trash 5. ◯ in yard 6. ◯ Outside premises 7. ◯ Into sink or tub 8. ◯ Thrown into waterway 9. ◯ At the well 10. ◯ Don’t know
72b		11. ◯ Thrown elsewhere (specify):
Annex RANAS questions for latrine use		
73. How often do you use a latrine for urinating and defecating? - Never - Seldom - Sometimes - Most of the time - (Almost) always		
74. Do you feel there is a risk that you contract diarrhea if you always use a latrine? - Yes - No		
75. Do you feel there is a risk that you contract diarrhea if you never use a latrine? - Yes - No		
76. Does it take an effort to always use a latrine? - Yes - No		
77. Will using a latrine prevent you from getting diarrhea? - Yes - No		
78. Is it time consuming to clean a latrine? - Yes - No		
79. Do you think maintaining a latrine is expensive? - Yes - No		
80. Do you like using a latrine? - Yes - No		
81. Does it bother you to defecate in the open? - Yes - No		
82. Does it bother you to use a latrine? - Yes - No		
83. Do you think a latrine smells? - Yes - No 84. How many people in your household always use a latrine? - (Almost) nobody - Some of them - Half of them - Most of them - (Almost) all of them		
85. How many people in your community always use a latrine? - (Almost) nobody - Some of them - Half of them - Most of them - (Almost) all of them		
86. People who are important to you, do they think you should always use a latrine? - Yes - No		
87. People in your community, do they think you should always use a latrine? - Yes - No		
88. Do you feel a responsibility to yourself to always use the latrine? - Yes - No		
89. Which items are critical for a good latrine? - Clean walls - Clean surface - Sanplat - Walls - Door - Roof - Pit - Personal cleaning items - Respondent didn’t know - Other		
90. Would you use the latrine rather than open defecation, even when you are in a hurry, for example because your child is crying? - Yes - No		
91. Would you repair the latrine if it is damaged after rainy season? - Yes - No		
92. Imagine you have stopped using the latrine for a few days, for example because the latrine was damaged. Are you confident that you will start using the latrine again? - Yes - No		
93. Do you plan to clean and maintain your latrine at regular times? - Yes - No		
94. Do you make sure to always have a latrine available, for instance when you are in the field or visiting a neighbor? - Yes - No		
95. Do you plan to use a latrine when you are working in the field? - Yes - No		
96. How often does it happen that you want to use the latrine, but it is too dirty for use? - Never - Seldom - Sometimes - Most of the time - (Almost) always		
97. How often do you forget to use the latrine? - Never - Seldom - Sometimes - Most of the time - (Almost) always		
98. Are you committed to always using a latrine, even if this requires planning, cleaning, maintenance, and a little financial burden for repairs? - Yes - No		
Section 5: Sanitation, part 2 • If the household has its own latrine at their household, skip to question 80 • If the household does NOT have its own latrine, continue with question 73		
99	How much time does it take on average to get to the place you defecate?	| ___ | ___ |___| minutes
100	Do you want to have your own household latrine?	1. ◯ Yes (go to question 102) 2. ◯ No (go to question 101)
101a	Why do you not want to have a latrine? • Circle all responses.	Expensive (go to question 103)
101b		Materials not available (go to question 103)
101c		Satisfied with neighbor's latrine or shared latrine (go to question 103)
101d		Does not see benefit in having a latrine (go to question 103)
101e		Culturally unacceptable (go to question 103)
101f		Other (specify): (go to question 103)
102a	Why do you want to have a latrine? • Circle all responses.	Dignity, appearance, or social status
102b		Health-related reasons
102c		Time or distance spent walking to a latrine
102d		Safety from others when walking to a shared latrine or the bush
102e		Other (specify):
103a	What is your reason for not having a latrine? • Circle all responses.	Expensive
103b		Construction materials are not available in the market
103c		Latrine slabs are not available in the Market
103d		There is no one with technical capacity
103e		They do not see any benefits in having their own latrine
103f		There are higher priorities than a latrine
103g		Culturally unacceptable
103h		Other (specify):
104	Do you plan on building a latrine by the start of the next rainy season?	1. ◯ Yes (go to question 105) 2. ◯ No (go to question 106)
105a	Who will pay for the construction of your latrine? • Circle all responses.	Me, my family, or my household members
105b		Neighbor or friend
105c		Community members, or chief
105d		An NGO or outside organization
105e		The government, HEF, or other officer of water or health
105f		The latrine will not cost any money
105g		Other (specify):
Section 6: Hygiene		
106	Have you been taught about hygiene and handwashing?	1. ◯ Yes (go to question 107) 2. ◯ No (go to question 108)
107a	Who taught you about hygiene or handwashing? • Circle all responses.	CBE students
107b		Children or other students
107c		Health Extension Workers
107d		Village Health Teams
107e		Other (specify):
108a	Please mention all of the occasions when you wash your hands. • Do not read the answers. • Circle all responses. • After they have finished responding, ask “are there any more times?” • If the respondent indicates that (s)he does not know, do not probe for additional responses.	Before eating
108b		After eating
108c		Before praying
108d		Before breastfeeding or feeding a child
108e		Before cooking or preparing food
108f		After defecation/urination
108g		After cleaning a child who has defecated/changing a child’s nappy
108h		When my hands are dirty
108i		After cleaning the toilet or potty
108j		In the morning
108k		Other (specify):
109	Do you and your family members wash your hands at all of these times?	1. ◯ Always 2. ◯ Sometimes 3. ◯ Never
RANAS questions for hand washing/Practice of handwashing		
110. The person who prepares the food, does he/she wash their hands with soap and water before preparing it? - Never - Seldom - Sometimes - Most of the time - (Almost) always		
111. Do you wash your hands with soap and water after toilet use? - Never - Seldom - Sometimes - Most of the time - (Almost) always		
Risk factors		
112. What are the direct consequences of diarrhea to you? - Loose, watery stool / frequent toilet use - Loss of water/ salt from the body - Loss of weight/ underweight - Fever, weakness, body/ stomach ache - Don’t know - Other		
113. Imagine a child has diarrhea. What are the indirect or long-term consequences of diarrhea for that child? - High medical costs - School Absence - Stunting (Low height for age) - Malnutrition (Difficulty in taking op food nutrients) - Don’t know - Other		
114. What are typical ways you can get diarrhea? - Don't wash hands - Consume contaminated food (germs, rotten) - Consume contaminated drinking water - Don’t know - Other		
115. Do you feel there is a risk that you will contract diarrhea if you always wash hands with soap and water after contact with stool? - Yes - No		
116. Do you feel there is a risk that you will contract diarrhea if you never wash your hands with soap and water after contact with stool? - Yes - No		
117. Do you feel there is a risk that you will contract diarrhea if you always wash your hands with soap and water before handling food? - Yes - No		
118. Do you feel there is a risk that you will contract diarrhea if you never wash your hands with soap and water before handling food? - Yes - No		
119. Would there be an impact on your daily life if you contracted diarrhea? - Yes - No 120. Would there be an impact on your family’s income if you contracted diarrhea? - Yes - No		
Attitude factors		
121. Does it take an effort to always wash hands with soap and water? - Yes - No		
122. Will always washing hands with soap and water prevent you from getting diarrhea? - Yes - No		
123. Is it time-consuming to always wash hands with soap and water? - Yes - No		
124. Do you think safe water is expensive? - Yes - No		
125. Do you think soap is expensive? - Yes - No		
126. Do you like washing hands with soap and water? - Yes - No		
127. Does it bother you if you cannot always wash your hands with soap and water after toilet use? - Yes - No		
128. Does it bother you if you cannot always wash your hands with soap and water before preparing/handling food? - Yes - No		
129. Do you like the feeling of clean hands? - Yes - No		
Norm factors		
130. How many people in your household always wash hands with soap and water after toilet use? - (Almost) nobody - Some of them - Half of them - Most of them - (Almost) all of them		
131. How many people in your community always wash hands with soap and water after toilet use? - (Almost) nobody - Some of them - Half of them - Most of them - (Almost) all of them		
132. People who are important to you, do they think you should always wash your hands with soap and water? - Yes - No		
133. People in your community, do they think you should always wash your hands with soap and water? - Yes - No		
Ability factors		
134. Do you feel a responsibility to yourself to always have clean hands? - Yes - No		
135. In which situations is it critical to wash hands with water and/or soap? - After defecating - After cleaning a child’s bottom - After other contact with stool - Before breastfeeding a child - Before feeding a child - Before preparing food - Before handling drinking water - Before eating - Don’t know - Other		
136. Would you wash your hands with soap and water, even when you are in a hurry, for example, because your child is crying. - Yes - No; 137. Would you wash your hands with soap and water, even when your (tippy) tap is broken? - Yes - No		
138. Imagine you have stopped washing hands for a few days, for example, because there was no water or soap for hand washing. Are you confident that you will start washing hands again? - Yes - No		
Self-regulation factor block		
139. Do you plan to install a hand washing facility with running water? - Yes - No		
140. Is the hand washing station located at a fixed location? - Yes - No		
141. Will you keep the soap close to the hand washing station? - Yes - No		
142. Do you make sure to always being able to wash your hands? - Yes - No		
143. Imagine that you are in a hurry, for example because your child is crying. Do you have a plan to ensure that you will still be able to wash your hands? - Yes - No		
144. Does it happen that you want to wash your hands with soap and water, but there is no water at home? - Never - Seldom - Sometimes - Most of the time - (Almost) always		
145. How often do you forget to wash your hands after toilet use or before handling food? - Never - Seldom - Sometimes - Most of the time - (Almost) always		
146. Are you committed to always washing your hands with soap and water even if this requires planning, maintenance of a hand washing facility, and a little financial burden for buying soap, water or for repairs? - Yes - No		
Section 7&8: Observations for Open Defecation Free Status • If the household has a latrine and handwashing station that you are allowed to observe, continue with question 147 • If the household does NOT have a latrine, or has NOT allowed you to see their latrine, skip to question 164		
147	Observe: presence of feces in the vicinity	Yes No
148	Observe: Presence of latrine with superstructure	Yes No
149	Observe: Maintenance: Is there a hole cover/lid?	1. ◯ No hole cover present 2. ◯ Hole cover defective, broken, or not used 3. ◯ Hole cover placed over hole and is tight fitting
150a	Observe Latrine use: Is there visibly used anal cleansing material in the latrine or in the pit?	1. ◯ Yes 2. ◯ No
150b	Observe: Are there fresh or recent feces evident in the pit? Use flash light	1. ◯ Yes 2. ◯ No
150c	Observe: if waterborne system, water is available	Yes No
150d	Observe: smell in a toilet/latrine	Yes 2. No
151	Observe: Are there flies present inside the latrine?	1. ◯ Yes – more than 10 flies 2. ◯ Yes – less than 10 flies 3. ◯ No
152a	Observe: Construction: What are the floor and slab made of?	1. ◯ Sticks or branches and dirt or clay 2. ◯ Wooden boards 3. ◯ Concrete 4. ◯ Plastic
152b		5. ◯ Other (specify):
153	Observe: Construction: What are the walls made of?	1. ◯ Walls are completely deteriorated or collapsed 2. ◯ Walls are made of a temporary material such as straw or palm leaves 3. ◯ Walls are made of durable materials such as wooden boards, concrete, or adobe
154	Observe: Construction: What is the quality of the door?	1. ◯ Door is absent, or door does not close properly. 2. ◯ Door is present and can be closed.
155	Observe: Construction: What is the quality of the roof?	1. ◯ No roof, or roof in complete disrepair with large gaps that offer no protection 2. ◯ Roof present but leaky 3. ◯ Roof present and provides protection from sun and rain
156	Observe: Maintenance: What is the quality of the slab?	1. ◯ Slab is significantly eroded, deteriorated to the point of being a safety concern. 2. ◯ Hole significantly eroded or other small gaps or cracks in slab. Not yet a safety hazard. 3. ◯ Slab more or less intact. No danger of children or adults slipping on uneven eroded surfaces, or of a foot or leg entering the pit through enlarged hole or other gaps in the slab.
157	Observe: Maintenance: What privacy does the latrine have?	1. ◯ User visible from outside (no walls, or walls do not provide privacy to user). 2. ◯ Cosmetic issues in need of repair, even though user is not visible from the outside. 3. ◯ Walls in sufficient repair to provide privacy.
158	Observe: Maintenance: How clean is the hole/opening area of the latrine?	1. ◯ Dry and clean 2. ◯ Dry but smeared with shit 3. ◯ Wet but no smeared shit 4. ◯ Wet and smeared with shit
Section 8: Washing Station Observations		
159	Observe: Is there a hand washing station inside the latrine or within 10 paces of the latrine?	1. ◯ Yes (go to question 160) 2. ◯ No (go to question 165)
160	Observe: Is there water at this hand washing station?	1. ◯ Yes (go to question 162) 2. ◯ No (go to question 163)
161a	Observe: What device is used for water at this handwashing station?	1. ◯ Tap 2. ◯ Tippy tap 3. ◯ Bucket 4. ◯ Wash basin
161b		5. ◯ Other (specify):
162a	Observe: Is there a handwashing material at this hand washing station inside/near the latrine? • Circle all responses	1. ◯ None 2. ◯ Soap 3. ◯ Detergent 4. ◯ Ash 5. ◯ Mud/sand
162b		6. ◯ Other (specify):
163	Observe: Does the washing station look like it has been recently used?	1. ◯ Yes 2. ◯ No
Section 9: Interactions		
164	Were you at the meeting when triggering/igniting happened in your community? • If the subject doesn’t understand the question, ask “did you participate in the community triggering/igniting for sanitation and hygiene?” • If the subject still doesn’t understand the question, mark “2 No”.	1. ◯ Yes (go to question 165) 2. ◯ No (go to question 166)
165a	Can you tell me what activities happened that day at the triggering/igniting? • Do not read the answers. • Circle all responses. • After they have finished responding, ask “were there any more activities?” • If the respondent indicates that (s)he does not know, do not probe for additional responses.	Visiting open defecation sites (transect walk)
165b		Visiting refuse dumps
165c		Community mapping (mapping houses, latrines, refuse sites, open defecation sites)
165d		Shit calculation (calculating the amount of shit produced by the community)
165e		Fecal-oral contamination discussion (shit flow)
165f		Glass of water demonstration (putting a stick or hair into feces then water)
165g		Food demonstration (putting food near feces with flies)
165h		Medical cost calculation (calculating the cost of illness, diarrhea, treatment)
165i		Community action planning
165j		Does not remember
165k		Other (specify):
166	Have you discussed sanitation or handwashing issues with any of your neighbors in the past 2 months?	1. ◯ Yes 2. ◯ No
167	Does your village or subcounty provide labor for households that cannot afford to build their own latrines?	1. ◯ Yes 2. ◯ No
168	Does your village or subcounty provide construction materials for households that cannot afford to build their own latrines?	1. ◯ Yes 2. ◯ No
169	In the last 2 months, have you visited your nearest health Centre?	1. ◯ Yes 2. ◯ No
170a	In the past 2 months, has a Health Extension Worker visited your house?	1. ◯ Yes 2. ◯ No
170b	In the past 2 months, has a Village Health Team member visited your house for a sanitation health talk?	1. ◯ Yes 2. ◯ No
171	In the past 2 months, has a health profession student from MUST visited your house to talk about sanitation or hygiene?	1. ◯ Yes 2. ◯ No

## References

[1] (2024). World Health Organization water, sanitation and hygiene strategy 2018-2025.. Water, Sanitation and Hygiene: Strategy 2018-2025.

[2] Ezbakhe F, Giné-Garriga R, Pérez-Foguet A (2019). Leaving no one behind: evaluating access to water, sanitation and hygiene for vulnerable and marginalized groups. Sci Total Environ.

[3] (2024). UNICEF. Progress on household drinking water, sanitation and hygiene 2000-2022: Special focus on gender. Avaialable at: https://data unicef org/resources/jmp-report-2023. Progress on Household Drinking Water, Sanitation and Hygiene 2000-2022: Special Focus on Gender.

[4] Vyas S, Srivastav N, Mary D (2019). Measuring open defecation in India using survey questions: evidence from a randomised survey experiment. BMJ Open.

[5] Mara D (2017). The elimination of open defecation and its adverse health effects: a moral imperative for governments and development professionals. J Water Sanit Hyg Dev.

[6] Crocker J, Geremew A, Atalie F, Yetie M, Bartram J (2016). Teachers and sanitation promotion: an assessment of community-led total sanitation in Ethiopia. Environ Sci Technol.

[7] Fuente D, Allaire M, Jeuland M, Whittington D (2020). Forecasts of mortality and economic losses from poor water and sanitation in sub-Saharan Africa. PLoS One.

[8] (2025). Goal 6: Ensure access to water and sanitation for all. https://www.un.org/sustainabledevelopment/water-and-sanitation/.

[9] (2012). A Practical Facilitation Handbook: CHAST - Children’s Hygiene and Sanitation Training.

[10] (2025). Participatory hygiene and sanitation transformation (PHAST). https://sswm.info/humanitarian-crises/urban-settings/hygiene-promotion-community-mobilisation/important/participatory-hygiene-and-sanitation-transformation-%28phast%29.

[11] Guiteras R, Levinsohn J, Mobarak AM (2015). Sanitation subsidies. Encouraging sanitation investment in the developing world: a cluster-randomized trial. Science.

[12] Venkataramanan V, Crocker J, Karon A, Bartram J (2018). Community-led total sanitation: a mixed-methods systematic review of evidence and its quality. Environ Health Perspect.

[13] Jenkins MW, Sugden S (2024). Jenkins MW, Sugden S. Rethinking sanitation: Lessons and innovation for sustainability and success in the new millennium. Human Development Report Office (HDRO), United Nations Development Programme …. Rethinking Sanitation: Lessons and Innovation for Sustainability and Success in the New Millennium.

[14] Routray P, Schmidt WP, Boisson S, Clasen T, Jenkins MW (2015). Socio-cultural and behavioural factors constraining latrine adoption in rural coastal Odisha: an exploratory qualitative study. BMC Public Health.

[15] Barnard S, Routray P, Majorin F, Peletz R, Boisson S, Sinha A, Clasen T (2013). Impact of Indian Total Sanitation Campaign on latrine coverage and use: a cross-sectional study in Orissa three years following programme implementation. PLoS One.

[16] McGranahan G, Mitlin D (2016). Learning from sustained success: how community-driven initiatives to improve urban sanitation can meet the challenges. World Dev.

[17] Kar K, Chambers R (2008). Handbook on Community-Led Total Sanitation.

[18] Ogendo KN, Kihara AB, Kosgei RJ, Tweya H, Kizito W, Murkomen B, Ogutu O (2016). Assessment of community led total sanitation uptake in rural Kenya. East African Med J.

[19] Zimba R, Ngulube V, Lukama C (2016). Chiengi district, Zambia open defecation free after 1 year of community-led total sanitation. Am J Trop Med Hyg.

[20] Gebremariam B, Tsehaye K (2019). Effect of community led total sanitation and hygiene (CLTSH) implementation program on latrine utilization among adult villagers of North Ethiopia: a cross-sectional study. BMC Res Notes.

[21] Pickering AJ, Djebbari H, Lopez C, Coulibaly M, Alzua ML (2015). Effect of a community-led sanitation intervention on child diarrhoea and child growth in rural Mali: a cluster-randomised controlled trial. Lancet Glob Health.

[22] Hammer J, Spears D (2016). Village sanitation and child health: effects and external validity in a randomized field experiment in rural India. J Health Econ.

[23] Siega-Sur JL, Woolley T, Ross SJ, Reeve C, Neusy AJ (2017). The impact of socially-accountable, community-engaged medical education on graduates in the Central Philippines: implications for the global rural medical workforce. Med Teach.

[24] Myers J (2019). Rural sanitation in Africa: challenges, good practices and ways forward. Frontiers of CLTS: Innovations and Insights 12.

[25] Harter M, Inauen J, Mosler HJ (2020). How does Community-Led Total Sanitation (CLTS) promote latrine construction, and can it be improved? A cluster-randomized controlled trial in Ghana. Soc Sci Med.

[26] Crocker J, Saywell D, Bartram J (2017). Sustainability of community-led total sanitation outcomes: evidence from Ethiopia and Ghana. Int J Hyg Environ Health.

[27] Bor D, Greer A (2003). Position paper on community-based education for health professionals. Educ Health.

[28] Mahoney S, Boileau L, Floridis J, Abi-Abdallah C, Lee B (2014). How social accountability can be incorporated into an urban community-based medical education program: an Australian initiative. Educ Health (Abingdon).

[29] Obol JH, Akera P, Ochola PA (2018). Community-based training of medical students is associated with malaria prevention and treatment seeking behaviour for children under 5 years in Uganda: a study of MESAU-MEPI COBERS in Uganda. BMC Med Educ.

[30] Hughes BO, Moshabela M, Owen J, Gaede B (2017). The relevance and role of homestays in medical education: a scoping study. Med Educ Online.

[31] Kristina TN, Majoor GD, van der Vleuten CP (2006). Comparison of outcomes of a community-based education programme executed with and without active community involvement. Med Educ.

[32] Okayama M, Kajii E (2008). Subjects of the training program related to the students' impressions and evaluations of community-based clinical training. Med Educ.

[33] Okayama M, Kajii E (2011). Does community-based education increase students' motivation to practice community health care?--a cross sectional study. BMC Med Educ.

[34] Atuyambe LM, Baingana RK, Kibira SP (2016). Undergraduate students’ contributions to health service delivery through community-based education: a qualitative study by the MESAU Consortium in Uganda. BMC Med Educ.

[35] Ndaruhutse GR (2013). Enhancing health service delivery through a university-local government partnership model, issues and experiences from Uganda. Commonw J Local Gov.

[36] Omotara B, Padonu M, Yahya S (2004). Assessment of the impact of community-based medical education of the University of Maiduguri on communities in three local government areas of Borno State, Nigeria: Community leaders′ perspectives. Educ Health.

[37] (2017). Republic of Uganda Ministry of Health Annual Health Sector Performance Report 2016/2017.

[38] (2019). Concept Note: Extension and Expansion of the Uganda Sanitation Fund Programme In: Division EH, editor. Safely Managed Sanitation Services in the Global Sanitation Fund.

[39] (2024). Republic of Uganda Ministry of Water and Environment. Ministry of Water and Environment Sector Performance Report. Water and Environment Sector Performance Report 2017.

[40] Crocker J, Saywell D, Shields KF, Kolsky P, Bartram J (2017). The true costs of participatory sanitation: evidence from community-led total sanitation studies in Ghana and Ethiopia. Sci Total Environ.

[41] Tyndale-Biscoe P, Bond M, Kidd R (2013). ODF sustainability study. FH Designs Australia: PLAN International. ODF Sustainability Study.

[42] Muriisa RK (2008). Decentralisation in Uganda: prospects for improved service delivery. Africa Dev.

[43] Pattanayak SK, Poulos C, Yang JC, Patil SR, Wendland KJ (2009). Of taps and toilets: quasi-experimental protocol for evaluating community-demand-driven projects. J Water Health.

[44] (2018). Rubaya Subcounty Sanitation Report.

[45] Rutterford C, Copas A, Eldridge S (2015). Methods for sample size determination in cluster randomized trials. Int J Epidemiol.

[46] Hayes RJ, Bennett S (1999). Simple sample size calculation for cluster-randomized trials. Int J Epidemiol.

[47] Amusa TO, Azeez KK, Olabode EA. (2023). KoboToolbox: Data collection on KoboCollect app. PARKS.

[48] Pattanayak SK, Yang JC, Dickinson KL (2009). Shame or subsidy revisited: social mobilization for sanitation in Orissa, India. Bull World Health Organ.

[49] Yeboah-Antwi K, MacLeod WB, Biemba G (2019). Improving sanitation and hygiene through community-led total sanitation: the Zambian experience. Am J Trop Med Hyg.

[50] Capps JM, Njiru H, deVries P (2017). Community-led total sanitation, open defecation free status, and ebola virus disease in Lofa County, Liberia. J Health Commun.

[51] Delaire C, Kisiangani J, Stuart K, Antwi-Agyei P, Khush R, Peletz R (2022). Can open-defecation free (ODF) communities be sustained? A cross-sectional study in rural Ghana. PLoS One.

[52] Okolimong CD, Ndejjo R, Mugambe RK, Halage AA (2020). Effect of a community-led total sanitation intervention on sanitation and hygiene in Pallisa District, Uganda. Am J Trop Med Hyg.

[53] Kouassi HA, Andrianisa HA, Traoré MB, Sossou SK, Nguematio RM, Djambou MD (2023). Factors influencing community-led total sanitation (CLTS) implementation abandonment before achieving open defecation-free (ODF) status: case study of the Central-Western region of Burkina Faso. Environ Sci Pollut Res Int.

[54] Sigler R, Mahmoudi L, Graham JP (2015). Analysis of behavioral change techniques in community-led total sanitation programs. Health Promot Int.

[55] Orgill-Meyer J, Pattanayak SK, Chindarkar N (2019). Long-term impact of a community-led sanitation campaign in India, 2005-2016. Bull World Health Organ.

[56] Hoo YR, Joseph G, Rivera R (2022). Strategic complements: poverty-targeted subsidy programs show additive benefits on household toilet purchases in rural Cambodia when coupled with sanitation marketing. PLoS One.

[57] Strasser R, Worley P, Cristobal F, Marsh DC, Berry S, Strasser S, Ellaway R (2015). Putting communities in the driver's seat: the realities of community-engaged medical education. Acad Med.

